# Exploring the performance of *Escherichia coli* outer membrane vesicles as a tool for vaccine development against Chagas disease

**DOI:** 10.1590/0074-02760220263

**Published:** 2023-05-22

**Authors:** María Elisa Vázquez, Andrea Cecilia Mesías, Leonardo Acuña, Joseph Spangler, Brenda Zabala, Cecilia Parodi, Meghna Thakur, Eunkeu Oh, Scott Allan Walper, Cecilia Pérez Brandán

**Affiliations:** 1 Consejo Nacional de Investigaciones Científicas y Técnicas, Universidad Nacional de Salta, Instituto de Patología Experimental Dr Miguel Ángel Basombrío, Salta, Argentina; 2 US Naval Research Laboratory, Center for Bio/Molecular Science & Engineering, Washington, DC, United States of America; 3 George Mason University, Fairfax, Virginia, United States of America; 4 US Naval Research Laboratory, Optical Science Division, Washington, DC, United States of America

**Keywords:** outer membrane vesicle, Trypanosoma cruzi, vaccine

## Abstract

**BACKGROUND:**

Vaccine development is a laborious craftwork in which at least two main components must be defined: a highly immunogenic antigen and a suitable delivery method. Hence, the interplay of these elements could elicit the required immune response to cope with the targeted pathogen with a long-lasting protective capacity.

**OBJECTIVES:**

Here we evaluate the properties of *Escherichia coli* spherical proteoliposomes - known as outer membrane vesicles (OMVs) - as particles with natural adjuvant capacities and as antigen-carrier structures to assemble an innovative prophylactic vaccine for Chagas disease.

**METHODS:**

To achieve this, genetic manipulation was carried out on *E. coli* using an engineered plasmid containing the Tc24 *Trypanosoma cruzi* antigen. The goal was to induce the release of OMVs displaying the parasite protein on their surface.

**FINDINGS:**

As a proof of principle, we observed that native OMVs - as well as those carrying the *T. cruzi* antigen - were able to trigger a slight, but functional humoral response at low immunization doses. Of note, compared to the non-immunized group, native OMVs-vaccinated animals survived the lethal challenge and showed minor parasitemia values, suggesting a possible involvement of innate trained immunity mechanism.

**MAIN CONCLUSION:**

These results open the range for further research on the design of new carrier strategies focused on innate immunity activation as an additional immunization target and venture to seek for alternative forms in which OMVs could be used for optimizing vaccine development.

Vaccines are one of the most successful medical advances to reduce morbidity and mortality from infectious diseases in modern times. Their fundamental role has been undoubtedly demonstrated by the elimination and reduction of several infectious diseases. It is estimated that over 2 million lives are saved every year due to their effectiveness^1^ and this statement becomes more evident than ever with the severe acute respiratory syndrome coronavirus 2 (SARS-CoV-2) pandemic outbreak in early 2020.^2^ It is widely known that the pathogens we defy are biologically diverse and have evolved different strategies to ensure their survival and persistence within their host. Therefore, each vaccine formulation and new medical technology may require a specific setup for the induction of a proper immune response.

New technologies are being investigated in the hope to improve and develop innovative vaccine strategies. One of these novel platforms comes from the emerging field of extracellular vesicles (EVs); in particular, Gram-negative bacterial outer membrane vesicles (OMVs) have shown a striking potential.^3^ OMVs are bilayered-lipid membrane, spherical nano-structures that are naturally released from Gram-negative bacteria into the extracellular environment. These vesicles are enriched with major outer membrane proteins and may contain adhesins, sulfatases and proteases that facilitate host-epithelial cells internalization.^4^ Further, OMVs are endowed with three valuable features for new vaccine-platform design: (1) they can transport antigens in a stable manner, attached to their surface or within the luminal space; (2) they are rapidly phagocytosed by antigen-presenting cells, allowing an efficient display of the derived peptides; and (3) they contain many pathogen-associated molecular patterns (PAMPs) which, through their binding to specific receptors, play a fundamental role in stimulating innate and promoting adaptive immune response. In fact, OMVs can simultaneously trigger antigen-specific humoral and T-cell-mediated responses.^5^ ^,^ ^6^ ^,^ ^7^ ^,^ ^8^ An additional attribute of these vesicles is the possibility of manipulating their protein cargo by genetic engineering, paving the way for equipping these structures with a heterologous antigenic repertoire.^9^ On the whole, the inherent characteristics of OMVs make them an attractive and versatile bioactive tool for the development of novel vaccines against a wide variety of pathogens.^3^

It is certainly feasible to exploit OMVs-mediated immunogenicity for parasitic infection-prophylaxis or even for immunotherapy purposes. In the case of diseases caused by trypanosomatid parasites, such as leishmaniasis, sleeping sickness, and Chagas disease (CD), it has not been possible to devise a totally protective and long-lasting vaccine to date, although diverse and exhaustive studies have been done.^10^ ^,^ ^11^ ^,^ ^12^ ^,^ ^13^ CD is not only a neglected tropical illness affecting approximately 6-7 million people worldwide, but also a chronic sickness that may result in disability in ~40% of the infected subjects due to cardiac, digestive, neurological, or mixed alterations.^14^ It is caused by the protozoan parasite *Trypanosoma cruzi,* a uniflagellated intracellular parasite with the ability to silently invade almost any nucleated mammalian cell and efficiently evade the host’s immune response.^15^ A broad spectrum of formulations has been evaluated over the years, including whole/attenuated parasites, recombinant proteins, viral and bacterial vectors, as well as DNA vaccines.^16^ ^-^ ^23^ However, protection achieved by these means is still very limited. Since OMVs are considered as promising nanoparticulate delivery systems for vaccination with superior advantages for antigen display and adjuvanticity,^24^ ^,^ ^25^ we evaluated this alternative platform for the development of a new vaccine for CD. The approach consists of evaluating *in vivo* the performance of native OMVs derived from a non-pathogenic *Escherichia coli* strain or genetically engineered OMVs expressing a *T. cruzi* antigen. To the best of our knowledge, these findings provide the first evaluation of the utility and effectiveness of native and engineered OMVs as a potential immunogenic delivery platform for trypanosomatid infections upholding future studies for improvement of their antigen-cargo and delivery.

## MATERIALS AND METHODS

*Ethic statement* - All animal protocols adhered to the National Institutes of Health (NIH) ‘‘Guide for the care and use of laboratory animals’’ and were approved by the School of Health Sciences and by the Ethical Committee of the Universidad Nacional de Salta, Argentina (Nº 311/18).

*Bacterial strains, media, and culture conditions* - Assembled plasmids were transformed into Chemically Competent *E. coli* 10β Cells (New England Biolabs, Ipswich, MA, USA) or DH5α for colony screening and plasmid maintenance. Sequence-confirmed plasmids were purified and transformed into *E. coli* BL21[DE3] (New England Biolabs, USA) for protein expression and OMVs production. Bacterial cultures were grown in Luria-Bertani (LB) or Terrific Broth (TB) media (ThermoFisher Scientific, Waltham, MA, USA) following the manufacturer’s protocols for media preparation. Cultures maintaining functional plasmids were grown in antibiotic-containing media to keep selective pressure (kanamycin sulfate, 50 µg/mL or ampicillin, 100 µg/mL, Sigma Aldrich, Kenilworth, NJ, USA).

*Plasmids design for OMVs-Tc24 production and rTc24 expression* - Plasmids for OMVs localization of Tc24 (termed OMVs-Tc24) were constructed using methods described before.^26^ The initial construct was made by amplifying the *Tc24* gene (TcCLB.507891.38) from genomic DNA isolates of *T. cruzi* CL Brener strain using Q5 Hotstart High-Fidelity Polymerase (New England Biolabs, USA) in 5 µL reactions consisting of 1 ng genomic DNA, 0.25 mM dNTP, 0.5 µM primers [Supplementary data (Table)], and 0.02 U/µL Q5 polymerase in 1X Q5 reaction buffer, following the manufacturer’s protocol to generate overlap regions of 15-18 bp. The pET28 plasmid backbone containing the lpp’OmpA fusion partner for directed insertion to the outside portion of the bacterial outer membrane was amplified following a similar protocol.^27^ ^,^ ^28^ After initial amplification of the plasmid backbone and *Tc24* gene, the polymerase chain reaction products were diluted 1:100 in water for repeat amplification of 17 cycles before combining 0.5 µL each to 50 µL Chemically Competent 10β Cells (New England Biolabs, USA) for heat shock transformation, following the manufacturer’s protocol. Transformants were selected by plating 10β cells on LB agar with 50 µg/mL kanamycin. The resulting positive transformants were grown for plasmid extraction and DNA sequencing to verify construction. Sequence-confirmed plasmids were transformed into chemically competent *E. coli* BL21[DE3] cells, following the manufacturer’s protocols. After recovery, aliquots were plated in antibiotic-containing medium for selection of positive transformants.

To construct the Tc24-expressing plasmid, the full-length *Tc24* sequence was cloned into the pRSET-A plasmid (ThermoFisher Scientific, USA). Genomic *T. cruzi* CL Brener DNA (100 ng) was used as a template for PCR assays. The reaction was performed in a Verity thermal cycler (ThermoFisher Scientific, USA). The amplification mixture contained 10 pmol of each Tc24-specific primer [Supplementary data (Table)], 1.5 mM MgCl_2_, 200 mM dNTPs, PCR buffer, 2.5 U *Taq* polymerase (ThermoFisher Scientific, USA), and water to a final volume of 25 µL. After denaturation at 94ºC for 5 min, thermal cycling was performed with 35 cycles of 94ºC for 30 s, followed by 58ºC for 30 s, and then 72ºC for 30 s. Reactions were finished by an extension at 72ºC for 5 min. The *Tc24* gene was cloned between BglII and EcoRI restriction sites and fused in the N-term to a 6× histidine-tag in pRSET-A plasmid. The pRSET-A-Tc24 construction was then transformed into *E. coli* DH5α competent cells, grown in LB containing 100 µg/mL ampicillin, and purified with the Qiagen Plasmid Maxi Kit (Germantown, MD, USA), according to the manufacturer’s specifications. The identity of the construct was confirmed by automatic sequencing in an ABI/Hitachi Genetic Analyzer 3130 belonging to the CERELA-CONICET sequencing facility (Tucumán, Argentina).

*Generation of nOMVs and OMVs-Tc24* - The following methods apply to both native (nOMVs) and antigen-loaded (OMVs-Tc24) OMVs. For simplicity, we refer to only OMVs-Tc24 samples. All nOMVs were purified from BL21[DE3] bacteria, therefore, antibiotics were excluded from growth media. OMVs purification was done as follows: single colonies were picked from LB-kanamycin plates maintaining *E. coli* BL21[DE3] cells containing the pET28-lpp’OmpA-Tc24 plasmid and transferred to 5 mL of LB medium plus antibiotic (kanamycin sulfate, 50 µg/mL). Cultures were incubated at 37ºC, shaking at 250 rpm overnight (12-16 h). Following incubation, 100 µL of the overnight culture was transferred to 50 mL TB plus kanamycin in a 250 mL baffled flask. Cultures were incubated at 37ºC, 250 rpm for 3 h or until reaching the mid-log stage as assessed by measurement of optical density at 600 nm (OD_600_). Expression of recombinant Tc24 and OMV-packaging was induced at OD_600_ = 0.8 - 1.0 by the addition of 0.5 µM Isopropyl β-D-1-thiogalactopyranoside (IPTG, Sigma-Aldrich, USA). To limit variability between controls and samples, IPTG was also included for untransformed bacteria cultures. Induced cultures were incubated overnight at 30ºC and 250 rpm.

Cultures were transferred to 500 mL polypropylene centrifuge bottles and centrifuged at 4,000 ×*g* in a fixed angle rotor to pellet bacterial cells. OMVs-containing supernatants were decanted to new 500 mL bottles and centrifugation repeated. Transfer and centrifugation were repeated until there was no visible bacterial pellet (2-4 times). The clarified supernatant was then filtered through a 0.45 μm PES membrane using a vacuum filtration flask to remove residual cells and large particulates. Filtered culture media was transferred to clear ultracentrifuge tubes (Beckman Coulter, Brea, CA, USA) and centrifuged at 109,000 ×*g* at 4ºC for 90 min using a swinging bucket rotor. The supernatant was decanted and centrifugation tubes inverted to allow residual media to drain. OMVs were resuspended in 1 mL phosphate buffered saline (PBS) 1X.^27^ ^,^ ^28^

*Characterization of nOMVs and OMVs-Tc24* - Purified OMVs were quantitated via dynamic light scattering (DLS) and nanoparticle tracking instrumentation and software analysis tools, as described in detail elsewhere.^29^ ^,^ ^30^ Briefly, DLS was used to measure hydrodynamic size and sample concentration (particle numbers *per* milliliter). To do that, the synthesized OMVs samples were diluted to 10 times with PBS 1X to make 2 mL colloidal solution. Then, the samples were transferred into a cuvette and measurements were recorded on a ZetaSizer™ Ultra instrument equipped with a HeNe laser source (λ = 633 nm) (Malvern Instruments Ltd., Worcestershire, UK) and analyzed using Dispersion Technology Software (Malvern Instruments Ltd.). For particle concentration measurement, we used three different angles (front, mid, and back scattering) based on the protocol of the manufacturer. The measurement was repeated three times and the average number was presented as the measured hydrodynamic size (or Z-average) and sample concentration.

Total protein was determined via a bicinchoninic protein assay (BCA, ThermoFisher Scientific, USA), following the manufacturer’s protocol. After quantitative analysis, samples were aliquoted to 1.5 mL microcentrifuge tubes and lyophilized for transport and storage as described before.^29^ ^,^ ^31^

**Fig. 1:**
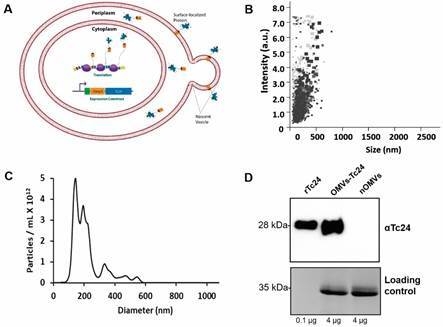
genetically engineered outer membrane vesicles (OMVs) are uniform vesicles able to display *Trypanosoma cruzi* Tc24 antigen efficiently. (A) Scheme of a nascent OMV from the outermost membrane of Gram-negative bacteria such as *Escherichia coli*. A recombinant gene circuit encoding a stimuli-responsive promoter (here via IPTG), lipoylation sequence (lpp’), membrane spanning domain (OmpA) and Tc24 was assembled and introduced to a non-pathogenic strain of *E. coli*. Activation of the gene circuit leads to protein synthesis, periplasmic targeting, and subsequent outer membrane insertion and OMV loading. (B) Particle intensity (in arbitrary units, a.u.) and size data was captured in triplicate over 90 s intervals and then converted to particle concentration and size. (C) Histogram demonstrating relative size and abundance of nOMVs and OMVs-Tc24 samples. (D) A representative Western blot image confirming the presence of Tc24 within OMVs-Tc24 content (4 µg per lane of total protein were loaded for both vesicle samples). Purified rTc24 protein (100 ng per lane) was used as a positive control. As expected, no presence of antigen Tc24 was detected in the nOMVs sample. Coomassie stained gel (cutout) is presented in the lower panel; a non-related vesicle protein band (~35 kDa) is shown as a loading reference.

*Recombinant Tc24 expression and purification* - Recombinant Tc24 (rTc24) was expressed from pRSET-A-Tc24 plasmid in *E. coli* BL21[DE3] grown in LB at 37ºC. When the culture reached an OD_600_ of ~ 0.6, rTc24 synthesis was induced with 0.5 mM IPTG (Sigma-Aldrich, USA) and the temperature was shifted to 28ºC. After 4 h of induction, cells were collected by centrifugation in PBS supplemented with 0.5 mg/mL of lysozyme (Sigma-Aldrich, USA), 0.01 mg/mL of DNAse (Sigma-Aldrich, USA), 0.5 mM phenylmethylsulfonyl fluoride (PMSF, Sigma-Aldrich, USA), and 1mM ß-mercaptoethanol (Bio-Rad, Hercules, CA, USA), lysed by three repetitive cycles of sonication (40% power, 3 × 45 s intervals), and one cycle of freezing and thawing. Subsequently, the lysate was centrifuged at 10,000 ×*g* at 4ºC for 30 min. The supernatant obtained was then purified through a Ni-NTA agarose cartridge (Qiagen, USA). After sorption of protein, the matrix was washed with 10 column volumes (CV) of buffer A (50 mM Tris-HCl, 300 mM NaCl, pH 8), followed by 5 CV of buffer A supplemented with 5 mM and 10 mM imidazole (GenBiotech, Buenos Aires, Argentina). Protein was eluted with 4 CV of buffer B (50 mM Tris-HCl, 300 mM NaCl, 500 mM imidazole, 10% glycerol, pH 7). Residual endotoxins from *E. coli* cell lysate were removed using a high capacity endotoxin removal resin (ThermoFisher, USA), following the manufacturer’s instructions. Protein was then dialyzed overnight in a cellulose tubing membrane (Sigma-Aldrich, USA) against 2 mM Tris-HCl, pH 7.4, and processed in a cold trap freeze-dryer (Heto Lab Equipment, AlerØd, Denmark). Finally, rTc24 was stored at -80ºC until use. The purity of the protein was assessed by sodium dodecyl sulphate-polyacrylamide gel electrophoresis (SDS-PAGE) [Supplementary data (Fig. 1)], and protein concentration was determined by the BCA method (ThermoFisher, USA). All SDS-PAGE images were acquired with ChemiDoc-It UVP2 imager (UVP, Upland, CA, USA).

*Tc24-antisera generation* - Polyclonal antibodies against rTc24 were raised by inoculating three mice (three-week-old males from the SWISS strain) by subcutaneous injection with 20 μg of recombinant protein adjuvanted with 15 µg of Quil-A (Invivogen, San Diego, CA, USA), followed by two booster doses of 10 μg of rTc24 each, supplemented in the same way at 38 and 50 days after the first immunization. Sera were collected 15 days after the last inoculation by cardiac puncture after deep terminal anesthesia.

*Western blot* - For SDS-PAGE, nOMVs and OMVs-Tc24 samples were homogenized by incubation in 100 μL lysis buffer (50 mM HEPES, 200 mM NaCl, and 1% NP-40, pH 7.4) containing a protease inhibitor cocktail (Promega, Madison, WI, USA). The vesicle lysates (4 μg of total protein *per* well) and rTc24 (100 ng *per* well) were resolved on a 10% polyacrylamide gel and then transferred to Immobilon-P PVDF membrane (Millipore, Burlington MA, USA. The membrane was blocked with 5% non-fat dry milk in 50mM Tris pH 7.0, 150mM NaCl (TBS) for 1 h, washed thrice with TBS - 0.05% Tween 20 (TBST), and then incubated for 1 h with anti-Tc24 antibody (1:1600-dilution in TBST). Then, the membrane was washed with TBST and incubated for 1 h with horseradish peroxidase-conjugated anti-mouse secondary antibody (Sigma-Aldrich, USA; 1:5000 dilution). The signal was developed and visualized by using an enhanced chemiluminescent detection reagent and hyperfilms (GE Healthcare Life Sciences, Pittsburgh, MA, USA). Coomassie blue staining was used as a loading control.

**Fig. 2:**
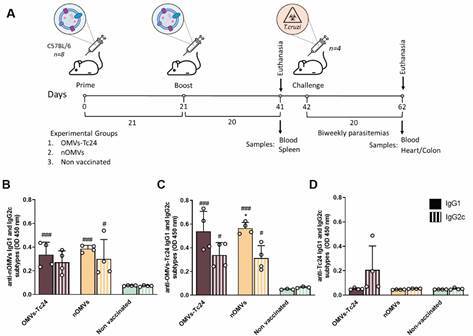
native outer membrane vesicles (nOMVs) and OMVs-Tc24 vaccinated animals elicited anti-OMVs specific humoral response. (A) Graphical representation of the immunization and challenge scheme. Animals were primed and boosted with nOMVs, OMVs-Tc24 or phosphate buffered saline (PBS) (non-vaccinated) three weeks apart. Twenty days after the second immunization, vaccine-induced IgG1 (solid bars) and IgG2c (striped bars) specificity was determined by enzyme-linked immunosorbent assay (ELISA) after incubating the serum samples with (B) nOMVs, (C) OMVs-Tc24 soluble lysates or (D) rTc24, respectively. Serum samples from non-vaccinated animals were used as negative controls. Data (mean ± SEM) are representative of three independent experiments (n = 4 mice *per* experimental group, duplicate observations *per* sample); significance is presented as # (non-vaccinated *vs.* vaccinated groups) or * (comparison between IgG1 and IgG2c within experimental groups). The p values of p ≤ 0.05, p ≤ 0.01, p ≤ 0.001 are annotated with one, two, and three symbols, respectively and were determined by one-way analysis of variance (ANOVA) with Tukey’s post-hoc test (comparison of multiple groups).

For semi-quantitative estimation of Tc24 load within OMVs, 1 μg total protein of OMVs-Tc24 sample was resolved along with a calibration curve of rTc24 (2.5 to 1280 ng *per* lane) through an SDS-PAGE. Then, the gel was transferred and Western blot performed in the same way as described before. The intensity signal of each band was quantified using ImageJ software^32^ and data was log-transformed to reach an optimal linear adjustment [Supplementary data (Fig. 2)].

*Animal model* - One-month-old C57BL/6 male mice were used throughout the immunization/challenge studies. Animals were housed in cages with up to 5 animals each and exposed to a 12 h light/dark cycle in a controlled temperature setting (25ºC) with free access to a standard laboratory chow diet and water. All the animals were bred in the Animal Facility of the Instituto de Patología Experimental and the Universidad Nacional de Salta, Argentina.

*Immunization protocol* - To evaluate OMVs immunization efficacy, groups of mice (n = 8 *per* group) were inoculated with two doses, three weeks apart, by subcutaneous injection with 50 µL total volume containing 15 µg of total protein of nOMVs or OMVs-Tc24. Additional reference group inoculated with PBS 1X (non-vaccinated) was included in the experiments. Before and after the infection challenge, blood was collected from the tail tip of mice under slight anesthesia in order to get serum samples for IgGs determination. Also, mice from each group were sacrificed by CO_2_ exposure for spleen removal and further splenocytes isolation and cytokine measurements. Colon and heart samples were also taken for qPCR analysis. For the record, infected mice were sacrificed at day 19 post-infection due to humanitarian termination.

*Specific IgGs detection and antibody-dependent complement-mediated lysis of trypomastigotes* - 96-well plates were coated with 1 µg/well of the target antigen (OMVs-Tc24, nOMVs) or 0.1 µg/well of rTc24 for measurement of specific IgG1 and IgG2c antibodies in sera from immunized animals by the enzyme-linked immunosorbent assay (ELISA) kit manufactured by Sigma-Aldrich as described elsewhere.^21^ For the antibody-dependent complement-mediated lysis assays, *T. cruzi* Sylvio X10/4 (ATCC 50823) culture-derived trypomastigotes were maintained and propagated by continuous *in vitro* passages in Vero cells (monkey fibroblast-like kidney cell line) supplemented with RPMI 1640 medium (Sigma Aldrich, USA), 5% FBS at 37ºC under a 5% saturated CO_2_ atmosphere.

Cell culture-derived trypomastigotes (5 × 10^5^/in 50 µL RPMI 1640/assay) were incubated with 50 µL of heat-inactivated sera (53ºC, 40 min) from vaccinated or control mice, at 37ºC for 1 h to allow antibody recognition. Next, non-decomplemented fresh human serum was added to a final 1:2 dilution for additional 3 h incubation. The remaining trypomastigotes were estimated by counting living, motile parasites in a Neubauer chamber, and the lytic ability of each serum was determined by comparison of the lysis obtained with and without specific external antibody source (immunized *versus* PBS-inoculated mice) in triplicate samples. Serum extracted from a chronically *T. cruz*i-infected mouse (> 3 months post-infection) was added as a positive external control.

*In vitro inhibition of cell infection by trypomastigotes* - To assess the experimental sera capacity to block parasite infection, Vero cells were seeded and cultured for 12 h in eight-well chambers (5 × 10^4^/well). Cell culture-derived trypomastigotes from Sylvio X10/4 strain were preincubated at 37ºC for 1 h with 50 μL of decomplemented sera from vaccinated or control mice. Again, a mouse serum sample from a *T. cruz*i-chronic infection was added as a positive control. Vero cells were then infected during a 3 h-incubation with pretreated trypomastigotes using a MOI of three. Parasites were then removed and washed cells remained in culture in RPMI 1640 5% FBS media, at 37ºC for 72 h. Slides were removed, washed with PBS, and cells were fixed with pure methanol. Then, glasses were flooded in freshly prepared, filtered 15% Giemsa solution (Biopur, Rosario, Argentina) for 12 min and washed with distilled H_2_O. The percentage of infected cells was estimated by counting total and infected cells *per* field; at least 10 fields *per* sample were analyzed. Representative images were obtained with a ZEISS Axio Scope.A1 optical microscope (×250 or ×400).

*Splenocytes cell culture and cytokine response* - Spleens from euthanized mice were removed for splenocytes isolation. Cells were resuspended in RPMI supplemented with 20 mM glutamine, 10% NaHCO_3_, and 10% FBS. The viability of cells was assessed by Trypan blue exclusion. Splenocytes (2 × 10^6^ cells/mL, in duplicate samples) were stimulated with 5 μg/mL of rTc24 or OMVs-Tc24 and cultured at 37ºC for 48 h and 5% CO_2_. Stimulation was also performed with 5 μg/mL of Concanavalin A (ConA, Sigma-Aldrich, USA) as a positive control. Cell culture medium was then collected and aliquots stored at -80ºC until their use for cytokine determinations. Measurement of IL-10, TNF-α and IFN-γ was performed using optEIA ELISA kits (BD Biosciences, San José, CA, USA) according to the manufacturer’s specifications.

*Flow cytometry* - Approximately 1 × 10^6^ splenocytes *per* sample were incubated in PBS supplemented with 1% FBS (PBS-FBS) and with the corresponding fluorochrome-conjugated specific monoclonal antibodies (CD3E FITC, CD8A PerCP-Cy5, CD4 PerCP-Cy5, CD127 Alexa 488, CD62L PE, CD44 PE-Cy7, BD Biosciences, USA) and incubated at 4ºC for 30 min in darkness. Cells were then washed with PBS and resuspended in PBS-FBS prior to their analysis. Cell acquisition of at least 10,000 events *per* sample of the lymphocyte gate was performed in a FACS Canto II flow cytometer (BD Biosciences, USA) and data were analyzed using the BD FACSDiva^TM^ software. The results are displayed as the percentage of each cell type in the total of spleen cells obtained.

*Trypanosoma cruzi challenge and parasitemias* - To evaluate the response to *T. cruzi* infection, mice were inoculated intraperitoneally with 500 blood trypomastigotes/mouse, belonging to Tulahuen strain (DTU *Tc*VI) and isolated from infected C57BL/6 mice. Challenge was done 20 days after the last immunization dose for short-term protection assessment. Biweekly, blood (10 μL) was drawn from the tail tip of mice under slight anesthesia, and the number of parasites *per* 100 fields (parasitemia) was recorded from fresh blood mounts under a light microscope (×400). Survival of the animals was recorded daily until they were sacrificed on day 19.

*Tissue parasite burden* - After the challenge, mice were sacrificed by CO_2_ overexposure. Heart and colon samples were taken for parasite DNA quantification. Total DNA from tissues (50 mg) was isolated using the ADN-Puriprep Highway nucleic acid kit (InbioHighway, Tandil, Argentina), according to instructions provided by the manufacturer. Real-time PCR was performed on a QuantStudio5 thermal cycler (AppliedBiosystems, Waltham, MA, USA) in a 20 μL reaction containing 100 ng of total DNA, 10 μL SensiFAST™SYBR® Hi-ROX Kit (BIOLINE, London, UK), and 1 µM SAT-*T. cruzi* specific oligonucleotides [Supplementary data (Table)] as described elsewhere.^33^ ^,^ ^34^ PCR cycling parameters were as follows: denaturing at 95ºC for 10 min, then 40 cycles of denaturing at 95ºC for 15 s, and annealing/amplifying for 30 s at 63ºC. Data were normalized to murine *tnf-*α amplification and analyzed using QuantStudio Design & Analysis Software from Applied Biosystem.

*Statistical analysis* - Data were analyzed by one‐way analysis of variance (ANOVA) followed by the Tukey *post hoc* test. Values are expressed as mean with standard errors of the mean (SEM) from at least two independent experiments with triplicate samples. Differences between experimental groups and control group, when considered significant, are shown as follows: *#* p ≤ 0.05, ## p ≤ 0.01, ### p ≤ 0.001. Differences among experimental groups when considered significant, are shown as follows: *p ≤ 0.05, **p ≤0.01, ***p ≤0.001.

## RESULTS

*Generation and characterization of nOMVs and OMVs-Tc24 displaying T. cruzi antigen* - All OMVs - native (nOMVs) or carrying the Tc24 antigen (OMVs-Tc24) - were obtained from *E. coli* BL21[DE3] cultures. To generate Tc24 antigen-carrying OMVs, a genetic construct was designed to achieve the surface localization of the *T. cruzi* antigen. As shown previously, a genetic construct comprising an N-terminal lipidation sequence (lpp’) and a bacterial recombinant transmembrane protein domain (OmpA) is sufficient for outer membrane localization and OMVs surface display of recombinant proteins.^28^ ^,^ ^31^ ^,^ ^35^ ^,^ ^36^ With this purpose, using an overlap PCR and cloning strategy,^31^ the Tc24 antigen was introduced in frame with the lpp’OmpA sequence and inserted into the lactose-inducible, bacterial expression vector pET28^36^ (Fig. 1A).

Since protein content of OMVs can vary widely depending on parameters like cell cycle, growth and induction conditions, duration of expression and others, purified OMVs samples were quantitated using total protein quantitative methods (BCA), dynamic light scattering (DLS), and nanoparticle analysis tools to obtain a comprehensive evaluation of the produced biomaterial. nOMVs and OMVs-Tc24 total protein yield was 270 μg/mL and 532 μg/mL, respectively. We used DLS to estimate hydrodynamic size and particle concentration of OMVs. After three repeated measurements, the average hydrodynamic size of OMVs was 115 ± 3 nm with a polydispersity of ~ 0.32, this second parameter is indicative of the heterogeneity of the sample based on size. In addition, the particle concentration obtained was estimated at 38 ± 7.3 nM in PBS. This measure was similar to our previous observation in which the vast majority of these particles fell within a range of 20-50 nm and 100-150 nm following a bimodal distribution of particle size.^30^ Similarly, the nanoparticle analysis indicated a particle size distribution between 80-150 nm, consistent with previous studies that used the same pET28 lpp’OmpA expression construct (Fig. 1B-C).

Continuing with the control of the nanovesicles generated, we evaluated the display of the heterologous Tc24 antigen in OMVs. For this purpose, we performed Western blot analysis over nOMVs and OMVs-Tc24 samples. According to the results observed, protein Tc24 was successfully delivered to bacterial vesicles (Fig. 1D). Of note, both rTc24 and OMVs anchored-Tc24 have ~28 kDa due to the 6× histidine-tag and the lpp’OmpA addition, respectively. In order to estimate antigen content within the harvested vesicles, we performed a semi-quantitative Western blot of OMVs-Tc24 along with the corresponding calibration curve of rTc24. Results obtained showed an estimation of ~23 ng of the recombinant antigen in 1 μg of total protein delivered to OMVs-Tc24, meaning around ~2% of the protein of interest among native repertoire [Supplementary data (Fig. 2)]. On the whole, quality control performed after production and purification of nOMVs and OMVs-Tc24 suggest that the nanoparticles obtained are within the expected parameters and represent a proper, although limited, transport for the desired *T. cruzi* antigen.

**Fig. 3:**
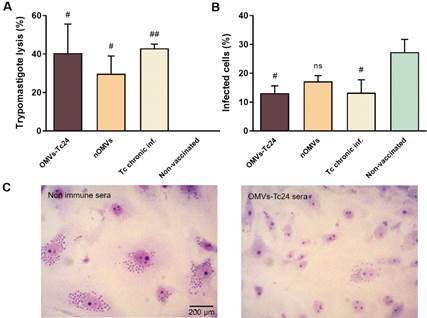
native outer membrane vesicles (nOMVs) and OMVs-Tc24 immunizations generate functional antibodies. (A) Activation of complement-mediated lysis of trypomastigote forms. Lysis percentage (%) was estimated by comparing the remaining number of motile trypomastigotes in samples pretreated with sera obtained from nOMVs and OMVs-Tc24 vaccinated *vs.* non-vaccinated animals (considered as 0%). (B) Percentage of *in vitro* infected cells after incubation with infective *Trypanosoma cruzi* forms pretreated with experimental sera [mice administered with nOMVs, OMVs or phosphate buffered saline (PBS)]. Data (mean ± SEM) are representative of two independent experiments with triplicate samples; each sera pool was composed of n = 4 animals *per* experimental group. Significance is presented as # (vaccinated groups *vs.* non-vaccinated). The values of p ≤ 0.05, p ≤ 0.01, p ≤ 0.001 are annotated with one, two, and three symbols, respectively and determined by one-way analysis of variance (ANOVA) with Tukey’s post-hoc test (comparison of multiple groups). *ns* = no significant differences among vaccinated groups (p > 0.05). (C) Representative images of Giemsa-stained infected cells after trypomastigote-pretreatment with serum from PBS-inoculated mice (left) and from OMVs-Tc24-administered animals (right) are shown; micrographs were obtained under a ×400 magnification factor.

*Native OMVs and OMVs presenting T. cruzi Tc24 antigen raise specific and functional antibodies* - After demonstrating that OMVs-Tc24 efficiently bears the selected antigen, we next inquired whether nOMVs along with this OMVs-Tc24 complex could induce a detectable specific humoral response. For this purpose, C57BL/6 mice were exposed to a prime and boost immunization scheme (Fig. 2A). Animals were inoculated with the OMVs formulations three weeks apart, and specific IgG1 and IgG2c antibody levels were measured 20 days after the last inoculation. Immunization of mice with nOMVs and OMVs-Tc24 induced both OMV-specific IgG1 and IgG2c antibodies at similar levels (Fig. 2B-C), whereas specific anti-Tc24 IgG2c antibodies were barely detectable in OMVs-Tc24-immunized mice (Fig. 2D). Interestingly, the levels of anti-OMVs specific IgG1 as well as IgG2c expand significantly after the administration of a third dose, not being the case for anti-Tc24 IgG subtype levels [Supplementary data (Fig. 3)].

Together, the results presented in Fig. 2 [and Supplementary data (Fig. 3)] suggest that immunization with nOMVs - as well as with OMVs-Tc24 - is capable of eliciting an anti-OMVs response in vaccinated animals (Fig. 2B-C), thus confirming the immunogenic nature of vesicles. Tc24 immunogenicity has been proved so far;^37^ ^,^ ^38^ ^,^ ^39^ however, even though OMVs-Tc24 are in fact carrying Tc24 - as corroborated in Fig. 1D - the amount of protein contained within engineered OMVs would not be enough to trigger a robust specific antibody response against the antigen of interest [Fig. 2D and Supplementary data (Fig. 3)].

Noticeably, OMVs-specific antibodies proved to be functional and neutralizing. Both nOMVs and OMVs-Tc24-induced antibodies succeeded in activating complement-mediated parasite lysis (Fig. 3A). This was evidenced by the incubation of infective *T. cruzi* forms with decomplemented experimental sera and the subsequent addition of an external complement source. The trypomastigote lysis observed after preincubation with sera from nOMVs- or OMVs-Tc24-inoculated mice displayed no significant difference in comparison with the lysis observed after preincubation with sera from chronically infected mice (OMVs-Tc24 41% and nOMVs 30% *vs*. cronically-infected 43%, p > 0.05, Fig. 3A). Furthermore, preincubation of cell culture-derived trypomastigote forms of Sylvio X10/4 strain with sera of OMVs-vaccinated animals significantly decreased *in vitro* invasion of non-phagocytic Vero cell monolayers compared with non-vaccinated animals (#, p ≤ 0.05, Fig. 3B-C). To conclude, immunizations with nOMVs or OMVs-Tc24 formulations trigger anti-OMVs specific antibodies able to neutralize trypomastigotes; thus, diminishing their invasion *in vitro* and facilitating complement-mediated lysis of parasites.

**Fig. 4:**
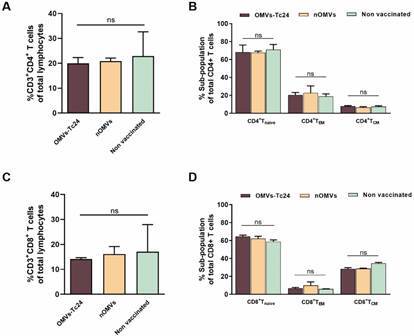
T cells profile after prime and boost with native outer membrane vesicles (nOMVs) and OMVs-Tc24. Nineteen days after boost, vaccinated mice were euthanized and freshly collected splenocytes were used for CD4^+^ and CD8^+^ T cell determination. Splenocytes were labeled with fluorescent-conjugated antibodies and analyzed by flow cytometry. (A, C) Splenic percentages of CD4^+^ and CD8^+^ T cells respectively. (B, D) CD4^+^ as well as CD8^+^ T cells were analyzed to distinguish *naïve* (CD44^lo^ CD62L^hi^), effector memory (T_EM_, CD44^hi^ CD62L^lo^), and central memory (T_CM_, CD44^hi^ CD62L^hi^) phenotypes. Data (mean ± SEM) are representative of duplicate observations *per* sample (n = 4 mice *per* group). Significance was determined by one-way analysis of variance (ANOVA) with Tukey’s post-hoc test (comparison of multiple groups). *ns* = no significant differences among groups, p > 0.05.

*Lack of variation in the memory phenotype of CD4* ^*+*^ *and CD8* ^*+*^ *T cells after OMVs prime and boost* - To determine whether immunization with nOMVs or OMVs-Tc24 generates a T cell profile able to confer protection against future virulent infection, we performed flow cytometry analyses of splenic cells obtained from vaccinated and control animal groups. From the total splenic lymphocyte population, the CD3^+^CD4^+^T cells constituted 20-21% in vaccinated animals (OMVs-Tc24 or nOMVs); a similar value (23%) was estimated in the non-immunized control group (Fig. 4A). Regarding the frequencies of CD4^+^T cells belonging to a *naïve* phenotype or associated with central (T_CM_) and effector (T_EM_) memory - determined by CD44^hi^, CD62L^hi^, and CD44^hi^, CD62L^low^ expression markers, respectively - no major changes were observed among the vaccinated groups in comparison with the control group (Fig. 4B). In reference to CD8^+^ T cell populations, the frequencies observed were relatively homogenous with no statistically significant differences among the experimental groups (Fig. 4C). Approximately 60% of the splenic CD8^+^ T cells showed a *naïve* phenotype in all groups whereas the frequencies of CD8^+^T_EM_ and CD8^+^T_CM_ subpopulations remained also similar among the analyzed groups (Fig 4D). Levels of IL-10, TNF-α and IFN-γ were measured in supernatants of stimulated splenic cells. No difference among groups was identified in terms of production of IL-10 and TNF-α while IFN-γ levels were undetectable (data not shown). Certainly, the obtained results differed from expected outcomes, thus suggesting that a higher antigen amount *per* dose would be necessary to trigger a robust T cell differentiation, at least under these experimental conditions.

**Fig. 5:**
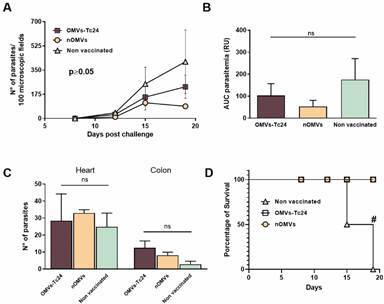
native outer membrane vesicles (nOMVs) and OMVs-Tc24 confer partial protection against *Trypanosoma cruzi* virulent infection. Animals inoculated with nOMVs, OMVs-Tc24, or non-vaccinated (n = 4) were challenged with 500 trypomastigotes from the Tulahuen strain, through intraperitoneal injection, 21 days after the last immunization dose. (A) Parasitemia curve obtained during the acute phase of the infection from 10 μL of blood taken twice a week. (B) Concentration-time curve (AUC) of the parasitemia curve (in relative units, RU). (C) Parasite equivalents in 100 ng of total DNA estimated by real-time qPCR amplification of SAT sequence at day 19 post-challenge. (D) Survival rates were monitored daily during the acute phase until animals from the control group started to die. Data are expressed as the mean ± SEM of three independent experiments. Significance was determined by one-way analysis of variance (ANOVA) with Tukey’s post-hoc test (comparison of multiple groups). Numeral represent statistical significance with respect to the non-vaccinated group. *ns* = no significant differences among groups, p > 0.05.

*nOMVs and OMVs-Tc24 immunization schemes are efficient in reducing circulating blood trypomastigotes* - Parasitemia measurement after an infection challenge is, without any doubt, a key parameter to evaluate an immunization strategy. To determine whether the immunizations with both OMVs formulations were able to protect mice from pathogen circulation and spread, 21 days after the last dose, animals were challenged with 500 bloodstream trypomastigotes of the Tulahuen *T. cruzi* strain, a widely known virulent strain that displays a characteristic tropism to heart and colon. As shown in Fig. 5A, all OMVs-containing formulations administered (nOMVs and OMVs-Tc24) correlated with a reduction in the number of circulating parasites in comparison with the non-vaccinated group. Furthermore, at the peak of parasitemia (19 days post-infection), all animals from the non-vaccinated control group started to succumb to death (Fig. 5D). To estimate the capacity of the vaccination approaches to reduce the total parasite blood load, we calculated the area under the parasitemia concentration-time curve (referred as AUC for area under the curve). Although there was no statistically significant difference among vaccinated groups, the one inoculated with nOMVs showed a considerable reduction in the total parasite load in comparison with the control group, while OMVs-Tc24-vaccinated animals displayed a quite moderate protection in parasite spread, not as evident as nOMVs (Fig. 5B). However, vaccinated and non-vaccinated mice exhibited high levels of *T. cruzi* DNA in heart and, to a lesser extent, in colon samples obtained during the acute phase of infection (Fig. 5C). Survival rate was also measured, reflecting that all OMVs-immunized animals were protected from lethal fate (Fig. 5D).

## DISCUSSION

During the last decades a huge expansion of the EVs field gained special consideration. These striking structures operate as cell-to-cell communication players associated with a plethora of functions and effects.^40^ ^,^ ^41^ Concerning the *Bacteria* domain, the vesicles secreted into the extracellular media fulfill important biological functions required for cell survival and bacteria-environment interactions.^42^ ^,^ ^43^ Within EVs, OMVs have acquired a new role as bioengineer tools envisaged for medical applications.^44^ ^,^ ^45^ ^,^ ^46^ ^,^ ^47^

As an illustration of the aforementioned applications, in the vaccinology area, many different studies aimed to characterize OMVs as nanovaccine devices.^3^ ^,^ ^48^ Indeed, there are already two licensed formulations against *Neisseria meningitidis serogroup b* (MenB), named Bexsero^®^ and VA-MENGOC-BC^®^, which are routine and safely administered in humans.^48^ Moreover, these vaccines were shown to offer additional moderate non-specific cross-protection against *N. gonorrhea*.^49^ Although the OMVs-based vaccine platform has been exhaustively exploited for several important diseases, there are, to date, no records of such approach evaluated for the treatment or prophylaxis of trypanosomatid infections. The present work represents the first exploratory assessment of *E. coli-*released OMVs as immunogenic carriers transporting *T. cruzi* antigens and as possible activators of a protective immune response aimed directly for the prevention of CD.

*Trypanosoma cruzi* Tc24 antigen has been widely analyzed as part of prophylactic^37^ ^,^ ^38^ ^,^ ^39^ and therapeutic^50^ ^,^ ^51^ strategies in mice and non-human primates undergoing acute and chronic infection. Thus, initiating the evaluation of OMVs as possible immunogens and nanocarriers of this candidate protein was an excellent opportunity to accomplish our goal of exploring their immune characteristics in a *T. cruzi* acute model of infection. As a first step of the present work, we analyzed mice’s specific humoral response after homologous OMVs-prime/boost vaccination. All nOMVs and OMVs-Tc24-immunized animals showed specific antibodies elicited by bacterial components, indicating that the dose/booster used and the route of immunization were accurate to induce an antibody response against the inoculated components. Still, this response could be potentiated if extra boosters are administered [Supplementary data (Fig. 3)]. The lack of anti-Tc24 specific antibodies relies undoubtedly on the poor antigen displayed within OMVs surface (~ 350 ng/dose) not able to properly activate a specific humoral response. Thus, our results pointed to OMVs as a double nature vaccine component, this meaning a structure able not only to act as an immunogenic component *per se* ^25^ ^,^ ^52^ but also to transport *T. cruzi* antigens, although vesicle´s antigen-load should be optimized.

Concerning the quality of the humoral response obtained, nOMVs and OMVs-Tc24 vaccination was able to trigger highly functional antibodies which proved to inhibit cell infection *in vitro*, as efficiently as total anti-*T. cruzi* antibodies from chronic-infected animals. Further, antibodies from nOMVs or OMVs-Tc24-vaccinated animals were also able to prime complement mediated-lysis similarly to serum from *T. cruzi* chronic-infected mice. Some molecular mimicry and cross-reaction mechanisms of the immune response elicited by *E. coli* components can be found in the literature.^53^ ^,^ ^54^ Yet, there is no record of any cross-reaction toward *T. cruzi* parasites. In an attempt to explain these unexpected results, one could speculate that nonspecific antibody-parasite interaction could have taken place under the present experimental conditions. Secondly, years ago Gunter and collaborators described natural IgM antibodies present in the *naive* repertoire of human and mice sera able to recognize *T. cruzi* Tc24 antigen, as part of an innate humoral mechanism.^55^ In our particular case, and even scarce, anti-Tc24 specific IgG antibodies were actually raised by OMVs-Tc24 complex inoculation, and almost certainly were associated with at least part of the complement lysis observed. However the presence of a nonspecific antibodies contribution effect cannot be ruled out and opens new tangential research questions, *i.e.* if OMVs inoculation could be capable of rising natural antibodies. Beyond all speculations, these results could imply that a humoral response of high quality, rather than quantity, is achieved by this carrier structure with no extra adjuvant supplementation.

A fundamental challenge in the development of a CD vaccine is the induction of a protective cell-mediated immunity and proper cytokine release.^56^ It is familiar that in *T. cruzi* infection, TNF-α, as well as IFN-γ, plays a major role in parasite clearance, while CD8^+^T, and CD4^+^T cells to a minor extent, arbitrate cytolytic activity through the release of perforin and granzymes.^57^ ^,^ ^58^ Overall, in our experiments, the total number of un-stimulated CD4^+^ and CD8^+^ memory T cells and their particular phenotype remained constant across the different groups. Also, the level of three crucial cytokines (IL-10, TNF-α and IFN-γ) were unaltered in the supernatants of stimulated splenocytes from mice receiving the nOMVs or OMVs-Tc24 formula (data not shown). Given the results obtained, which correlate with lack of total parasite clearance, we will aim to improve antigen cargo within OMVs formulation, so that they could adequately activate and enhance a robust protective immune response. In this sense, there are several studies supporting the idea that OMVs may trigger a balanced T_H_1/T_H_17 response^59^ and that this type of cellular response would be essential for a CD vaccine formulation.^60^ ^,^ ^61^ Taking this into consideration, OMVs-based vaccines may represent an interesting carrier to shape the response towards the proper immune phenotypes. In short, and beyond the type of immune response generated, what must be achieved through the administration of any potential immunogen is the control and reduction of the pathogen load. In this sense, all OMVs-immunized animals were successful in moderately control peripheral parasite load. However, when we explored parasite cargo in target organs in greater depth, we noted that protection was no longer maintained. Thus, our OMVs vaccine - whether carrying Tc24 antigen or not - showed the potential to stimulate a modest immune response able to cope by some means with circulating blood trypomastigotes burden but not to avoid the colonization of specific organs.

Surprisingly, mice vaccinated with nOMVs were also able to restrain *T. cruzi* infection, showing parasitemia levels similar to those observed in animals immunized with OMVs-Tc24. We could relate these findings to a non-specific protective response triggered by bacterial molecules present in OMVs which are recognized by Toll-like receptors (TLRs) such as TLR2, TLR4 and TLR9. These TLR agonists are emerging as immunomodulatory agents able to trigger a hyper-responsive reaction and confer cross-protection against related or even unrelated microorganisms upon a secondary heterologous exposure.^62^ These recently described mechanisms are part of the so-called trained (innate) immunity and are mainly grounded on a metabolic switch and epigenetic long-lasting modifications in myeloid, NK and type 2 innate lymphoid cells.^63^ ^,^ ^64^ In this way, several OMVs-based vaccines were able to confer cross-protection against non-related pathogens. Concerning protozoal infections, there are various studies in leishmaniasis and malaria evidencing non-specific beneficial effects of the Bacille Calmette-Guérin (BCG) vaccine, possibly the most characterized trained immunity inducer by now.^65^ Recently, a study involving a cohort of 19 healthy subjects exposed to a controlled *Plasmodium falciparum* infection showed that a previous administration of BCG vaccine favored the activation of NK cells and monocytes that correlated with a reduction in parasitemia.^66^ Finally and regarding CD, an epidemiological study conducted with 110 sero-positive patients has evidenced that those who had been previously vaccinated with BCG showed a better clinical evolution than non-vaccinated subjects.^67^ Certainly, this mechanism observed for OMVs deserves larger and deeper analysis, as in fact it could be capitalized in future formulation designs seeking for an innate immunity improvement, now defined as a new vaccine target.^68^

In brief, OMVs offer remarkable advantages since these vesicles proffer an easily scalable and cost-effective technology. Further, they can be lyophilized, stored, and transported without refrigeration;^69^ these attributes would offer an enormous impact on transportation costs and availability to marginal communities. It is well known that vaccines based on single antigens are not likely to be as effective as multi-antigen formulations, in this sense OMVs are susceptible to be fulfilled with several non-bacterial-antigens. In addition, OMVs themselves confer immunogenic properties, thus not requiring an extra adjuvant additive. Here we presented a first advance in the assessment of *E. coli*-OMVs as potential immunogens and nanocarriers for the delivery of *T. cruzi* antigens. To conclude, we consider that OMVs-based vaccines, along with their notable properties, deserve to be further characterized in view of the generation of an effective formulation for the prophylaxis or therapeutic management of CD and other neglected disorders.
