# Supplementary material for: Exploring the performance of Escherichia coli outer membrane vesicles as a tool for vaccine development against Chagas disease
Source: Mem Inst Oswaldo Cruz. 2023 May 22;118:e220263. doi: 10.1590/0074-02760220263 (PMC10207902; doi:10.1590/0074-02760220263)
Supplement: Supplementary file 1 [file 1678-8060-mioc-118-e220263-s.pdf]

TABLE

Sequences of the primers used for plasmid design. pRSET-A-Tc24 plasmid was constructed by amplifying *Trypanosoma cruzi* genomic DNA with primers Tc24-Rec-F/R. pET28-lpp'OmpA-Tc24 plasmid was constructed by amplifying *T. cruzi* genomic DNA with primers Tc24-F1/R1 and pET28-lpp'OmpA-PTE with primers pET28-F1/R3. Products were treated as described in methods. SAT-F/R and TNF-F/R: primers used for *T. cruzi* quantification by qPCR, amplify satellite parasite DNA sequence and TNF-α murine DNA sequence as normalizer gene, respectively

Primer name	Sequence (5'-3')
Tc24-Rec-F	GGAAGATCTTGGGTGCTTGTGGGTCGAAGG
Tc24-Rec-R	GGAGAATTCTCACGCGCTCTCCGGCACG
Tc24-F1	AGCATGGGTGCTTGTGGGTCG
Tc24-R1	GTGGTGGTGGTGGTGGTGCGCGCTCTCCGGCA
pET28f1	CACCACCACCACCACCACTGAGA
pET28r3	CGACCCACAAGCACCCATGCTCCCACCGCC
Lpp-F2	AACGCGAAAATTGATCAGGGCGGTGGTAGCGGTGGTGGCTCTGGT
Lpp-R2	GTGGTGGTGGTGGTGGTGGCTGCCGCCACCAGAGCCACCACC
SAT-F	GCAGTCGGCKGATCGTTTTCG
SAT-R	TTCAGRGTTGTTTGGTGTCCAGTG
TNF-F	TCCCTCTCATCAGTTCTATGGCCCA
TNF-R	CAGCAAGCATCTATGCACTTAGACCCC

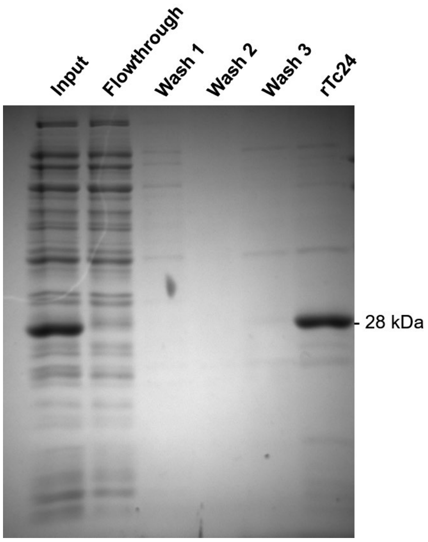

Fig. 1: purification process of rTc24. Representative sodium dodecyl sulphate-polyacrylamide gel electrophoresis (SDS-PAGE) of the Ni-NTA chromatography performed to obtain rTc24. Aliquots of the input, flowthrough, wash 1, 2 and 3 and rTc24 protein (~28 kDa) were loaded into a 15% SDS-PAGE.

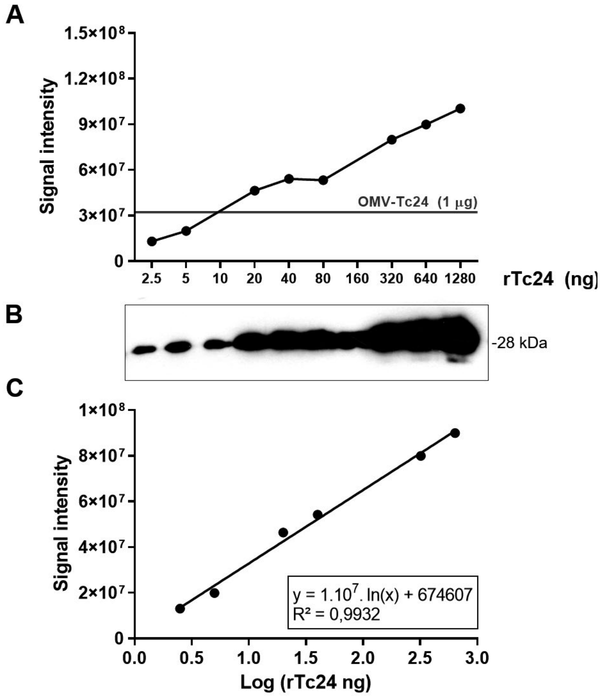

Fig. 2: semi-quantitative western blot of outer membrane vesicles (OMVs)-Tc24. (A) Each band intensity observed in a Western blot image was quantified (with arbitrary units) using ImageJ software and plotted for every rTc24 load. (B) Western blot of the rTc24 curve, with protein amounts varying from 2.5 to 1280 ng *per* lane were loaded and used to estimate the specific antigen-content in 1 µg of OMVs-Tc24 sample. (C) Variable was log-transformed in order to get a linear adjustment of the signal intensity *versus* recombinant protein load (R^2 = 0.99). Through this approach, the Tc24-content within 1 µg of OMVs-Tc24 was estimated at 23.55 ng.

CC BY

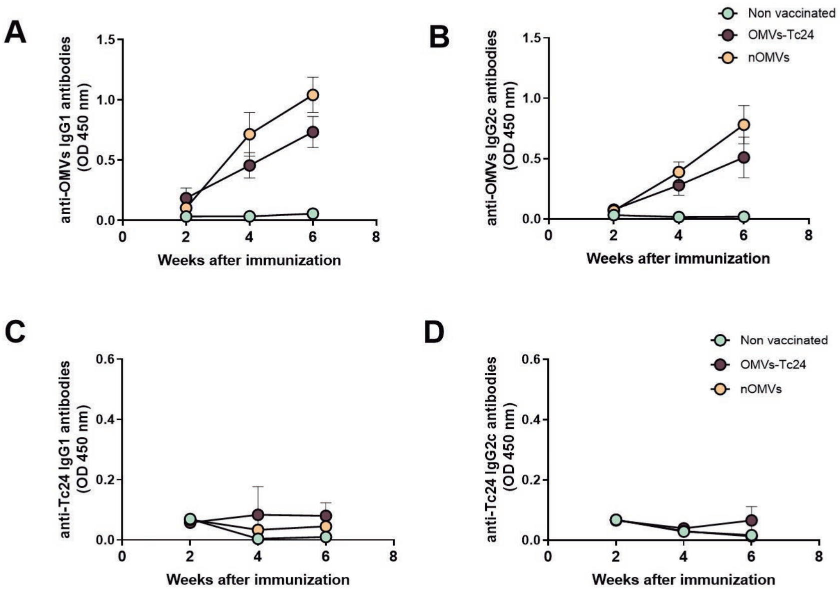

Fig. 3: rise of anti-outer membrane vesicles (OMVs) specific antibodies after a third immunization dose with native outer membrane vesicles (nOMVS) or OMVs-Tc24. Animals were primed and boosted twice with nOMVs, OMVs-Tc24 or phosphate buffered saline (PBS) (non-vaccinated) two weeks apart. Fifteen days after the third immunization, vaccine-induced IgG1 and IgG2c specificity was determined by enzyme-linked immunosorbent assay (ELISA). (A, B) Dynamic curve of anti-OMVs or (C, D) anti-Tc24 IgG antibody subtypes during the immunization period. Serum samples from non-vaccinated animals were used as negative controls. Data (mean ± SEM) are representative of two independent experiments (n = 4 mice *per* experimental group, duplicate observations *per* sample); significance at 6 weeks after immunization is presented as # (non-vaccinated vs. vaccinated groups). The p values of $p \leq 0.05$, $p \leq 0.01$, $p \leq 0.001$ are annotated with one, two, and three symbols, respectively and were determined by one-way analysis of variance (ANOVA) with Tukey's post-hoc test (comparison of multiple groups).
